# Explainable machine learning for long-term outcome prediction in two-center stroke patients after intravenous thrombolysis

**DOI:** 10.3389/fnins.2023.1146197

**Published:** 2023-02-22

**Authors:** Zheng Ping, She Huiyu, Li Min, Bai Qingke, Lu Qiuyun, Chen Xu

**Affiliations:** ^1^Department of Neurosurgery, Shanghai Pudong New Area People’s Hospital, Shanghai, China; ^2^The Center for Pediatric Liver Diseases, Children’s Hospital of Fudan University, Shanghai, China; ^3^Department of Neurology, Shanghai Eighth People’s Hospital, Shanghai, China

**Keywords:** cerebral infarction, machine learning, ischemic stroke, intravenous thrombolysis, modified rankin score, NIHSS

## Abstract

**Objective:**

Neurological outcome prediction in patients with ischemic stroke is very critical in treatment strategy and post-stroke management. Machine learning techniques with high accuracy are increasingly being developed in the medical field. We studied the application of machine learning models to predict long-term neurological outcomes in patients with after intravenous thrombolysis.

**Methods:**

A retrospective cohort study was performed to review all stroke patients with intravenous thrombolysis. Patients with modified Rankin Score (mRs) less than two at three months post-thrombolysis were considered as good outcome. The clinical features between stroke patients with good and with poor outcomes were compared using three different machine learning models (Random Forest, Support Vector Machine and Logistic Regression) to identify which performed best. Two datasets from the other stroke center were included accordingly for external verification and performed with explainable AI models.

**Results:**

Of the 488 patients enrolled in this study, and 374 (76.6%) patients had favorable outcomes. Patients with higher mRs at 3 months had increased systolic pressure, blood glucose, cholesterol (TC), and 7-day National Institute of Health Stroke Scale (NIHSS) score compared to those with lower mRs. The predictability and the areas under the curves (AUC) for the random forest model was relatively higher than support vector machine and LR models. These findings were further validated in the external dataset and similar results were obtained. The explainable AI model identified the risk factors as well.

**Conclusion:**

Explainable AI model is able to identify NIHSS_Day7 is independently efficient in predicting neurological outcomes in patients with ischemic stroke after intravenous thrombolysis.

## Introduction

Stroke is one of the common neurological diseases, among which ischemic stroke stays about 70–80% of adult stroke, and its incidence rate is rising every year (Benjamin et al., 2019). The average stroke incidence is 120–180/100,000/year, being greater for men (Thayabaranathan et al., 2022). The traditional treatments of ischemic stroke are mechanical thrombectomy and intravenous thrombolysis (Berkowitz et al., 2014; Alet et al., 2020), while the novel treatment strategy include cellular therapy and non-invasive brain stimulation (Richards and Cramer, 2023). However, due to the limited time window for the thrombolysis interventions, generally within 4.5 h and extended 6 h (Qingke et al., 2021), the early prediction of clinical outcomes in stroke patients is essential in post-stroke management.

The prognostic prediction models have been established thereafter (Jiang et al., 2021; Kerleroux et al., 2021). Although object prediction systems, such as ASTRAL (Michel et al., 2010), DRAGON (Wang et al., 2017), and THRIVE (Flint et al., 2014), have been reported to assess the efficiency of intravenous thrombolysis in ischemic stroke patients, most of these scales are based on traditional algorithms with limited clinical features. With recent developments in artificial intelligence, medical machine learning has produced several exciting findings (Jamin et al., 2021; Jayatilake and Ganegoda, 2021). Considering its extended impact on ischemic stroke management, machine learning (ML) models for outcome prediction in patients with intravenous thrombolysis were developed based on the comparison of clinical data according to the modified Rankin score (mRs) at 90 days after thrombolysis. Although some ML studies have identified the risk and protective factors in ischemic stroke (Livne et al., 2018; Heo et al., 2019; Lee et al., 2020; Yang et al., 2020; Ramos et al., 2022), however, the procedure of modeling is hard to be translated to the clinical session. Therefore, in our study, the predictive *p*-value in each model was further compared and validated in two external datasets. The explainable AI model was added to identify the risk factors as well.

## Materials and methods

We enrolled 930 ischemic stroke patients with thrombolytic therapy within 6 h of the stroke onset from July 2018 to June 2020 retrospectively in two stroke centers in the local hospital, whose age ranged from 18 to 80 years and head computed tomography (CT) scans showed no acute hemorrhage. Twelve patients with missing clinical data were excluded. For stroke patients with an onset within 4.5 h, rtPA was directly delivered, and for patients whose onset was 4.5–6 h, rtPA was not given until magnetic resonance imaging (MRI) showing new infarction area. The comprehensive treatments for these patients in two centers were consistent and based on both European stroke organizations (ESO) guidelines on intravenous thrombolysis (Berge et al., 2021) and Chinese guidelines.

The mRs system was used to evaluate the neurological outcome at 3 months after the thrombolysis for these patients, and mRs < 2 was considered as a favorable neurological outcome, while mRs 2–6 was poor outcome. This study was approved by the Ethical Board of Shanghai Pudong New Area People’s Hospital with a waiver of informed consent due to the retrospective nature of the study. Informed consent for intravenous thrombolysis was obtained from all patients.

### Machine learning algorithms

A total of 16 variables were included in the machine learning models establishment, including patient basic characteristics, time from onset to admission, history of previous diseases, NIHSS_Baseline, and NIHSS_Day7 (Supplementary Table 1). Hypertension, diabetes mellitus, and hyperlipidemia are all risk factors for stroke. Patient data regarding systolic pressure; diastolic pressure; and levels of blood glucose, triglyceride (TG), cholesterol (TC), and low-density lipoprotein (LDL) were included.

Ten machine learning algorithms were used in this study. For the random forest model, we calculated ROC curve with an AUC and an AP *p*-value as a reference for the logistic regression, SVM, RF and decisionTreeClassifier (DTC). Four ensemble learning algorithms, one unsupervised K-nearest neighbor model and one deep neural network model were applied. The predictive capability of machine learning models with several variables (sex; age; first onset of stroke; previous history of hypertension, diabetes, and hyperlipidemia; baseline systolic pressure; diastolic pressure; levels of blood glucose, TG, TC, and LDL; NIHSS score; for calculating the prediction score was investigated. Among the patient samples, 70% (*n* = 341) were randomly chosen as the training group and the remaining 30% (*n* = 147) were assigned to be the test group. We also included two external validation datasets. The detailed information of the validated sets were summarized in Supplementary Tables.

## Results

A total of 488 stroke patients with intravenous thrombolysis were included in this study. The mean age was 59.4 ± 6.2 years, and 25.82% of the patients were female. The basic characteristics between the stroke patients with high mRs and low mRs were compared in Supplementary Table 1.

Patients with higher mRs at 3 months after stroke were found to have greater systolic pressure (*P* = 0.034); levels of blood glucose (*P* = 0.002), TC (*P* = 0.001), LDL (*P* = 0.007), NIHSS_Baseline and NIHSS_Day7 (*P* < 0.001) compared to those with lower mRs, regardless of previous history of diabetes or hyperlipidemia (Supplementary Table 1).

### Comparison of prediction models for favorable outcomes

Favorable outcomes were found in 374 (76.6%) of the 488 patients. A total of 341 and 147 cases were separated into the training and testing data, respectively, with 20 features. The ML models were constructed based on the 10-fold cross validation. For the RF model, the mean f1 score was 0.975 in the training set, which was higher than the SVM method with 0.951 and higher than the logistic regression model at 0.943 (Table 1). However, for the DecisionTreeClassifier, it obtained a tree score of 0.83 (Supplementary Figures 1A, B), which was much lower compared to the RF, LR, and SVM models. The procedure for the decision tree analysis is shown by Graphviz in Supplementary Figure 1C. Consistently, in the testing dataset, the mean f1 score was 0.825 in RF, 0.653 in SVM and 0.777 in LR model (Table 2).

**TABLE 1:** Mean f1 scores in training in-house data.

Random forest mean	0.9749710144927537
SVM mean	0.951425892947632
Logistic regression mean	0.9427229437229437

**TABLE 2:** Mean f1 scores in testing in-house data.

RF Mean	0.824858757062147
SVM mean	0.6530612244897959
LR mean	0.7771428571428571

Four ensemble learning algorithm (with cross-verification network) was then applied and showed that the VotingClassifier, stacking model, bagging model, and boosting model obtained values of 0.891, 0.891, 0.884, and 0.871, respectively. An unsupervised learning model was then applied to predict the outcome as well. The decomposition with Kernel PCA was clustered with K-means with two components, and a seaborn was applied to draw the cluster map. Results showed that several clinical features could be clustered together (Supplementary Figure 2). The deep neural network model did not achieve higher accuracy score with 0.900) compared to the other ML models. And, the performance of the multiple-entry model had an accuracy of 0.927 (95% CI, 0.910–0.941) with a *p*-value loss of 0.5594 (Supplementary Figure 3). Therefore, we chosed the RF, LR, and SVM models with top three predictive score for further analysis.

### Model optimization of superparameter with grid research

We then assess the quality of each model, and showed the accuracy score of the RF model was 0.909 (Table 3). After adjusting the superparameter with grid research for RF [n_estimators = (64,100,128,200,300), max_features = (2,3,5,7,9), bootstrap = (True, False)], we chose the bootstrap, max_features = 5 and n_estimators = 200, then we increased the accuracy score further at 0.915 (Table 4).

**TABLE 3:** Random forest model quality before optimization.

	Precision	Recall	F1-score	Support
0	0.97	0.91	0.94	261
1	0.76	0.90	0.82	81
Accuracy			0.91	342
Macro avg	0.86	0.91	0.88	342
Weighted avg	0.92	0.91	0.91	342

Accuracy score: 0.9093567251461988.

**TABLE 4:** Random forest model quality after optimization.

	Precision	Recall	F1-score	Support
0	0.96	0.93	0.94	261
1	0.79	0.88	0.83	81
Accuracy			0.92	342
Macro avg	0.87	0.90	0.89	342
Weighted avg	0.92	0.92	0.92	342

Accuracy score: 0.9153046783625731; F1 score: 0.8304093567251462.

For the logistic model, when the superparameter was optically set at C with 10 and penalty at 12 [penalty = (’7’,’9’,’11’,’12’,’14’), C = (0.001, 0.01, 0.1, 1, 10, 100)], the accuracy score could be increased to from 0.886 to 0.900 (Tables 5, 6).

**TABLE 5:** LR model quality before optimization.

	Precision	Recall	F1-score	Support
0	0.95	0.90	0.92	261
1	0.72	0.84	0.78	81
Accuracy			0.89	342
Macro avg	0.84	0.87	0.85	342
Weighted avg	0.89	0.89	0.89	342

Accuracy score: 0.8859649122807017.

**TABLE 6:** LR model quality after optimization.

	Precision	Recall	F1-score	Support
0	0.95	0.91	0.93	261
1	0.75	0.85	0.80	81
Accuracy			0.90	342
Macro avg	0.85	0.88	0.86	342
Weighted avg	0.90	0.90	0.90	342

Accuracy score: 0.8976608187134503; F1 score: 0.7976878612716762.

For the SVM model [“C”: (0.1, 1, 10, 100, 1,000), “gamma”: (1, 0.1, 0.01, 0.001, 0.0001), “kernel”: (“rbf”)], after optimizing with C = 10, gamma = 0.1 and kernel = rbf, the accuracy score from 0.851 to 0.889, respectively (Tables 7, 8).

**TABLE 7:** SVM model quality before optimization.

	Precision	Recall	F1-score	Support
0	0.88	0.93	0.91	261
1	0.73	0.59	0.65	81
Accuracy			0.85	342
Macro avg	0.80	0.76	0.78	342
Weighted avg	0.84	0.85	0.85	342

Accuracy score: 0.8508771929824561.

**TABLE 8:** SVM model quality after optimization.

	Precision	Recall	F1-score	Support
0	0.94	0.91	0.93	261
1	0.74	0.83	0.78	81
Accuracy			0.89	342
Macro avg	0.84	0.87	0.85	342
Weighted avg	0.89	0.89	0.89	342

Accuracy score: 0.8888888888888888; F1 score: 0.7790697674418605.

**FIGURE 1:**
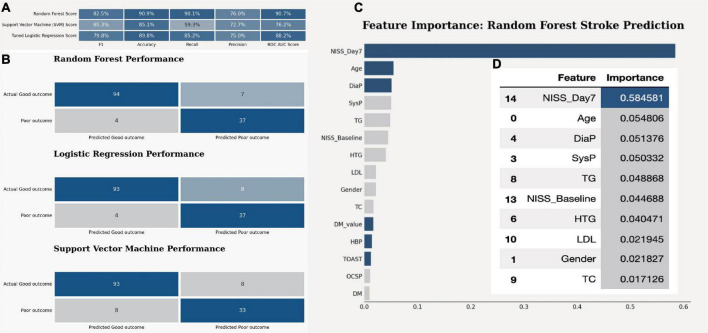
The model quality comparison among three ML models. **(A)** The F1 score and accuracy score in three models in the testing dataset. **(B)** The confusion matrix for three ML models in the testing dataset. **(C)** The bar plot shows the feature importance in random forest model. **(D)** The bar quantification for the feature importance in random forest model.

After optimizing the models with best parameters, the prediction ability of all models in the testing dataset has been increased a little bit as well (Figure 1A). Then we showed the confusion matrix in the testing dataset with three ML models (Figure 1B). We further listed the feature importance of most important factors in the RF model, which indicated that both NIHSS_Day 7 and NIHSS_Baseline were the most important factors in predicting the neurological outcome (Figures 1C, D).

### Explainable AI models

**FIGURE 2:**
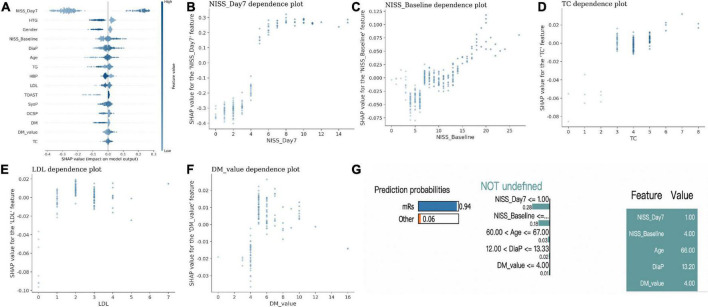
The explainable AI model in the outcome prediction in IS. **(A)** The SHAP *p*-value of each factor to differentiate the outcome in IS. **(B–F)** The scatter plot between each specific factor and its SHAP *p*-value. **(G)** One example of patient shows lower NIHSS_Day 7, lower NIHSS_Basline, lower age, DiaP and DM-*p*-value had a higher probability of good outcome with LIME model.

Next, we applied the explainable AI model and showed the SHAP *p*-value of each factor to see its impact in the RF model. Again, we found NIHSS_Day7 has the most critical impact on outcome compared to other factors (with a distinct SHAP *p*-value, Figure 2A). Meanwhile, NIHSS_Basline, TC, LDL, and DM_*p*-value were also risk factors with high SHAP feature *p*-value, which indicated that higher NIHSS, high expression of TC, LDL, and blood sugar level suggested the poor outcome (Figures 2B-F).

In addition, the LIME model also demonstrated one example with lower NIHSS_Day7, lower NIHSS_Basline, lower age, DiaP and DM-*p*-value had a higher probability of good outcome (Figure 2G).

**FIGURE 3:**
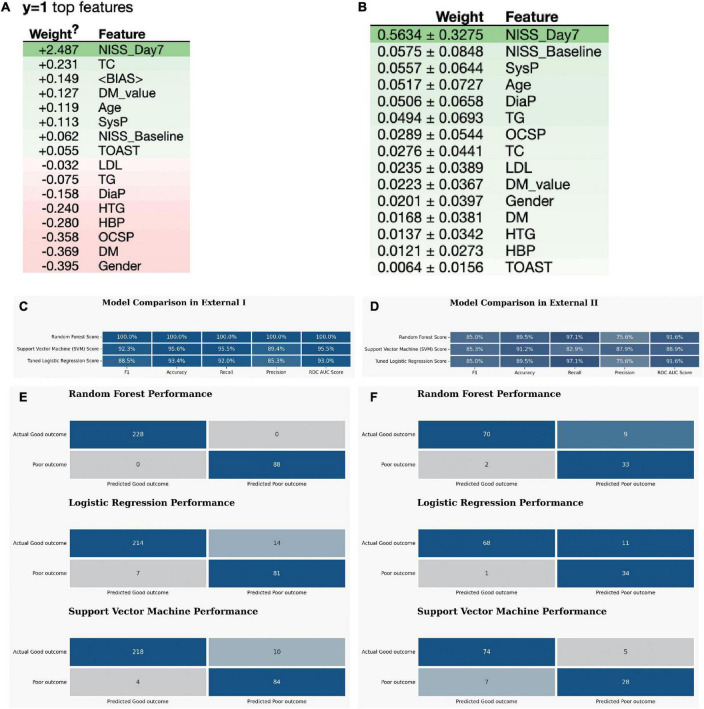
The external validation of the optimized three ML models. **(A,B)** The eli5 model shows the weight of each feature in the RF model. **(C)** The model quality comparison in the external dataset I. **(D)** The model quality comparison in the external dataset II. **(E)** The confusion matrix for three models in the external dataset I. **(F)** The confusion matrix for three models in the external dataset II.

At last, the eli5 model listed the weight of each feature in the RF model, and both NIHSS_Day7, NIHSS_Baseline and metabolic parameters: TC, TG, DM_*p*-value have a high impact on the outcome (Figures 3A, B).

### External validation

To validate the predictive ability of these machine-learning model in stroke after intravenous thrombolysis, the other external datasets from the other hospital including 316 stroke patients (control group) and 114 patients with wake-up stroke (WUS group) were used for validation. All these stroke patients had intravenous rt-PA as well. The demographic variables were compared between the stroke patients with high mRs and low mRs is shown in the Supplementary Tables 2, 3 for the two external datasets. Patients with higher mRs at 3 months after stroke were found to have greater levels of LDL, NIHSS_Baseline and NIHSS_Day7 compared to those with lower mRs (BOLD with Blue color in Supplementary Table 2) in External Data I, regardless of whether patients had previous hyperlipidemia.

Favorable outcomes were observed in 231 (73.1%) of the 316 control patients (Supplementary Table 2). For the External I, the predictive accuracy of the RF model was 100%. This is higher than that of the SVM, which obtained a score of 92.3%, and LR score at 88.5% (Figure 3C). The confusion matrix also showed that the RF model performed best in the External I (Figure 3E). The risk factor in External I group was baseline and NIHSS_Day7 score (Supplementary Tables 4–6).

In addition, we did the correlational analysis between the risk factors and found NIHSS_Day7 had the highest correlation with mRs at 0.84 in External Data I (Supplementary Figures 4, 5).

In External data II group, we found the favorable outcomes were observed in 76 (66.7%) of the 114 WUS patients (Supplementary Table 3). The predictive power of the logistic regression model was 89.5% (Figure 3D). This is equal to that of the RF, which obtained a score of 89.5% as well, but lower than the SVM score *p*-value (91.2%). The confusion matrix also indicated that both RF and SVM performed well (Figure 3F). The risk factor in External II group was NIHSS_Baseline and NIHSS_Day7 and DM_*p*-value (Supplementary Tables 7–9).

Furthermore, we did the correlational analysis between the risk factors and found NIHSS_Day7 had the highest correlation with mRs at 0.96 in External Data II (Supplementary Figures 6, 7).

### Comparison of the NIHSS parameters for the prediction of outcomes

Of the 488 patients, 374 (76.6%) patients had favorable outcomes. As NIHSS_Baseline, NIHSS_Day3, and NIHSS_Day7 were all correlated with the mRS score at 3 month, we further compared the predicting ability among them and we found NIHSS_Day7 has the highest AUC *p*-value (AUC = 0.953) to predict the mRS *p*-value, followed by NIHSS_ Day3 (AUC = 0.930) and NIHSS_Baseline (AUC = 0.687). The similar results were found in the external verification groups (Supplementary Figures 8A–C). Meanwhile, when we combined all NIHSS_Baseline, NIHSS_Day3, and NIHSS_Day7, we found that the AUC *p*-value in three groups were all more than 0.9, which indicated a strong prediction ability (Supplementary Figures 8D–F).

## Discussion

This study identified the benefit of explainable machine learning models for accurately predicting neurological outcomes in acute stroke patients treated with intravenous thrombolysis and further validated with external datasets. Machine learning modules have been reported to predict long-term outcomes in stroke patients, and deep neural networks can improve the long-term outcome prediction in patients with ischemic stroke (Heo et al., 2019). Heo et al. (2019) have demonstrated 78% of stroke patients had favorable outcomes and the deep neural network can improve the prediction of long-term outcomes in ischemic stroke patients (0.888 vs. 0.839 from ASTRAL score). In our studies, we had 16 inputs and the accuracy of RF model reached 90.9% as well. Among 10 ML models. We chose three for further analysis with the top three accuracy: RF, LR, and SVM compared to DTC, KNN, DNN, and four ensemble learning models. For simplicity, only a few significant variables with their coefficients roughly calculated were analyzed in the traditional machine learning model like logistic regression. From the respect of this, it can be concluded that prediction accuracy of the RF is better than traditional machine learning models, such as LR Model (accuracy at 89.8% in the testing dataset and 94.3% in the training dataset) (Yoshimura et al., 2018). Nevertheless, some factors may influence clinical outcomes of stroke and might have an effect on prediction as well, which is reflected from our results and external verifications. We therefore improved the accuracy and f1 score of these three models after optimization with super parameters. However, the theoretical background underlying improved performance is unknown.

Therefore, the explainable AI model such as: SHAP model, LIME model and eli5 model were applied to translate the AI into clinical session. From the SHAP model, we found NIHSS_Day7 has the most critical impact on outcome compared to other factors; meanwhile, NIHSS_Basline, TC, LDL and DM_*p*-value were also risk factors with high SHAP feature *p*-value, which indicated that higher NIHSS, high expression of TC, LDL, and blood sugar level suggested the poor outcome. Most importantly, we found NIHSS_Day7 is also an independent risk factor for mRs at 3 months (with the highest correlational co-efficiency, Supplementary Figure 5), which is quite consistent with a current study published in Stroke (Mistry et al., 2021). Mistry et al. (2021) reported that 24 h NIHSS was the strongest predictor of 90-day mRs outcomes for stroke patients with endovascular therapy. However, AUC *p*-value for 24-h NIHSS to predict the outcome in their study is 0.855, which is less than our prediction accuracy in both RF model (0.909) and LR model (0.898). Even so, the feasibility of applying acute or subacute NIHSS for outcome prediction with machine learning models in stroke patients needs to be verified in future studies with larger populations in multi-centers.

A recent study by Monteiro et al. (2018) focused on the baseline NIHSS score, glucose level, systolic and diastolic blood pressure, age, and NIHSS score (7 days) to predict the neurological outcome in stroke patients and found that the AUC of predicting models increased with more clinical features. Most clinical features were similar to those observed in this study, which indicated that the machine learning-based prediction is largely dependent on the number of features, however, Monteiro et al. (2018) did not include plasma lipids in stroke patients. As regulating hyperlipidemia is a Class IIB evidence in stroke management (Winstein et al., 2016), it is critical to include lipid markers in the prediction model.

There are some limitations to be addressed in this study. First, this was a two-center project with external validation from other groups, as the variables used as inputs to the machine learning algorithms were mostly different in most cases and different centers. It would be hard to do an horizontal comparison directly. However, compared to previous studies, the ASTRAL score was 0.839 (Michel et al., 2010), which is smaller compared to ours; this prediction ability might be influenced slightly according to the variables and be adjusted considering their availability when incorporating data from baselines. Second, the 16 clinical features were selected according to the shared terms in both centers. It would be ideal to add more clinical characteristics in future studies, such as brain radiomics, routine blood tests: such as white blood cells counts, neutrophils counts and lymphocytes. However, our model did not seem to provide the reference *p*-value form thrombolysis time window. Future studies are required to increase the clinical feature including time from stroke to thrombolysis (DNT) and Onset to therapy (OTT); Last but not least, all NIHSS_Baseline, NIHSS_Day3, and NIHSS_Day7 were able to predict the mRS scores at 3 months post-onset, and the NIHSS_Day7 holds the strongest prediction ability among them. Considering that machine learning models can be self-taught with additional data, the aforementioned results are subject to be improved and are promising.

## Conclusion

This study demonstrated absolute NIHSS_Day7, as a continuous variable, is an independent prediction factor for 90-day functional outcomes and machine learning algorithms, with explainable AI models can improve the neurological outcome prediction for ischemic stroke patients after intravenous thrombolysis, which provides potential targets for clinical intervention.

## Data availability statement

The original contributions presented in this study are included in this article/Supplementary material, further inquiries can be directed to the corresponding author.

## Ethics statement

The studies involving human participants were reviewed and approved by the Ethical Board of Shanghai Pudong New Area People’s Hospital. The patients/participants provided their written informed consent to participate in this study.

## Author contributions

ZP and BQ designed the study and performed the bioinformatical analysis with Python (v 3.8.8) and wrote the draft. SH, LQ, CX, and LM collected the clinical data. All authors approved the submitted version.
