# Supplementary material for: Explainable machine learning for long-term outcome prediction in two-center stroke patients after intravenous thrombolysis
Source: Front Neurosci. 2023 Feb 22;17:1146197. doi: 10.3389/fnins.2023.1146197 (PMC9992421; doi:10.3389/fnins.2023.1146197)
Supplement: Supplementary file 1 [file Data_Sheet_1.docx]

**Supp Table 1. Clinical characteristics of patients with different mRs**

| Variable | level | Overall | Lower mRs | Higher mRs | p-value |
| --- | --- | --- | --- | --- | --- |
| n |  | 488 | 374 | 114 |  |
| Gender | M | 362 (74.18%) | 275 (73.53%) | 87 (76.32%) | 0.636 |
|  | F | 126 (25.82%) | 99 (26.47%) | 27 (23.68%) |  |
| Age (years) |  | 60.00 (52.00-67.00) | 60.00 (52.00-67.00) | 60.00 (53.25-67.00) | 0.447 |
| First Onset | With | 15 ( 3.07%) | 13 ( 3.48%) | 2 ( 1.75%) | 0.534 |
|  | Without | 473 (96.93%) | 361 (96.52%) | 112 (98.25%) |  |
| HBP | With | 176 (36.07%) | 140 (37.43%) | 36 (31.58%) | 0.304 |
|  | Without | 312 (63.93%) | 234 (62.57%) | 78 (68.42%) |  |
| SysP (kPa) |  | 20.80 (18.67-22.53) | **20.60 (18.67-22.50)** | **21.33 (19.50-22.67)** | **0.034** |
| DiaP (kPa) |  | 12.00 (10.67-13.33) | 12.00 (10.67-13.33) | 12.00 (10.97-13.33) | 0.117 |
| DM | With | 407 (83.40%) | 308 (82.35%) | 99 (86.84%) | 0.325 |
|  | Without | 81 (16.60%) | 66 (17.65%) | 15 (13.16%) |  |
| DM_value(mmol/L) |  | 5.30 (4.70-6.30) | **5.20 (4.60-6.10)** | **5.50 (4.93-7.07)** | **0.002** |
| HTG | With | 286 (58.61%) | 219 (58.56%) | 67 (58.77%) | 1 |
|  | Without | 202 (41.39%) | 155 (41.44%) | 47 (41.23%) |  |
| TG (mmol/L) |  | 1.50 (1.02-2.10) | 1.54 (1.02-2.12) | 1.40 (1.00-1.85) | 0.118 |
| TC (mmol/L) |  | 4.43 (3.78-5.08) | **4.34 (3.67-4.99)** | **4.72 (4.06-5.29)** | **0.001** |
| LDL (mmol/L) |  | 2.70 (1.73-3.30) | **2.60 (1.66-3.30)** | **2.90 (2.22-3.48)** | **0.007** |
| TOAST | 1 | 14 ( 2.87%) | 10 ( 2.67%) | 4 ( 3.51%) | 0.463 |
|  | 2 | 40 ( 8.20%) | 33 ( 8.82%) | 7 ( 6.14%) |  |
|  | 3 | 430 (88.11%) | 329 (87.97%) | 101 (88.60%) |  |
|  | 4 | 4 ( 0.82%) | 2 ( 0.53%) | 2 ( 1.75%) |  |
| OCSP | 1 | 353 (72.34%) | **264 (70.59%)** | **89 (78.07%)** | **<0.001** |
|  | 2 | 104 (21.31%) | **91 (24.33%)** | **13 (11.40%)** |  |
|  | 3 | 17 ( 3.48%) | **15 ( 4.01%)** | **2 ( 1.75%)** |  |
|  | 4 | 14 ( 2.87%) | **4 ( 1.07%)** | **10 ( 8.77%)** |  |
| Onset_Time (hour) |  | 2.00 (1.05-3.25) | 2.00 (1.01-3.20) | 1.96 (1.10-3.29) | 0.975 |
| DNT (hour) |  | 1.33 (1.00-1.83) | 1.33 (1.02-1.80) | 1.41 (1.00-2.00) | 0.305 |
| OTT (hour) |  | 3.50 (2.57-5.02) | 3.58 (2.57-5.00) | 3.50 (2.57-5.38) | 0.988 |
| NIHSS_Baseline |  | 7.00 (5.00-10.00) | **6.00 (5.00-9.00)** | **9.00 (6.25-13.75)** | **<0.001** |
| NIHSS_Day7 |  | 2.00 (1.00-5.00) | **2.00 (1.00-3.00)** | **8.00 (6.00-10.00)** | **<0.001** |

**Supp Table 2. Clinical features in different mRs groups in External Data I**


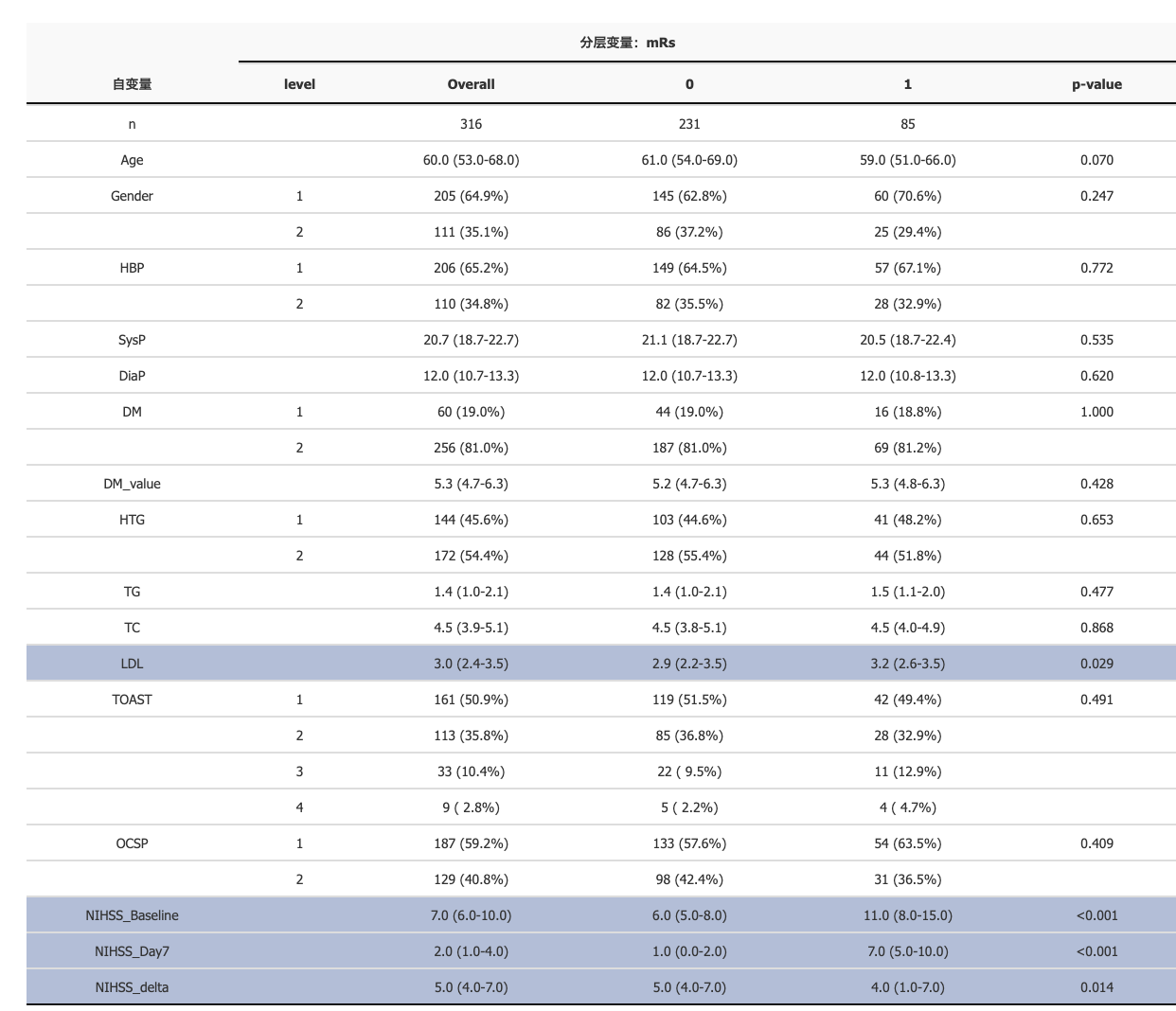


**mRs 0 group： mRs (0-1); mRs 1 group: mRs (2-6)**

**
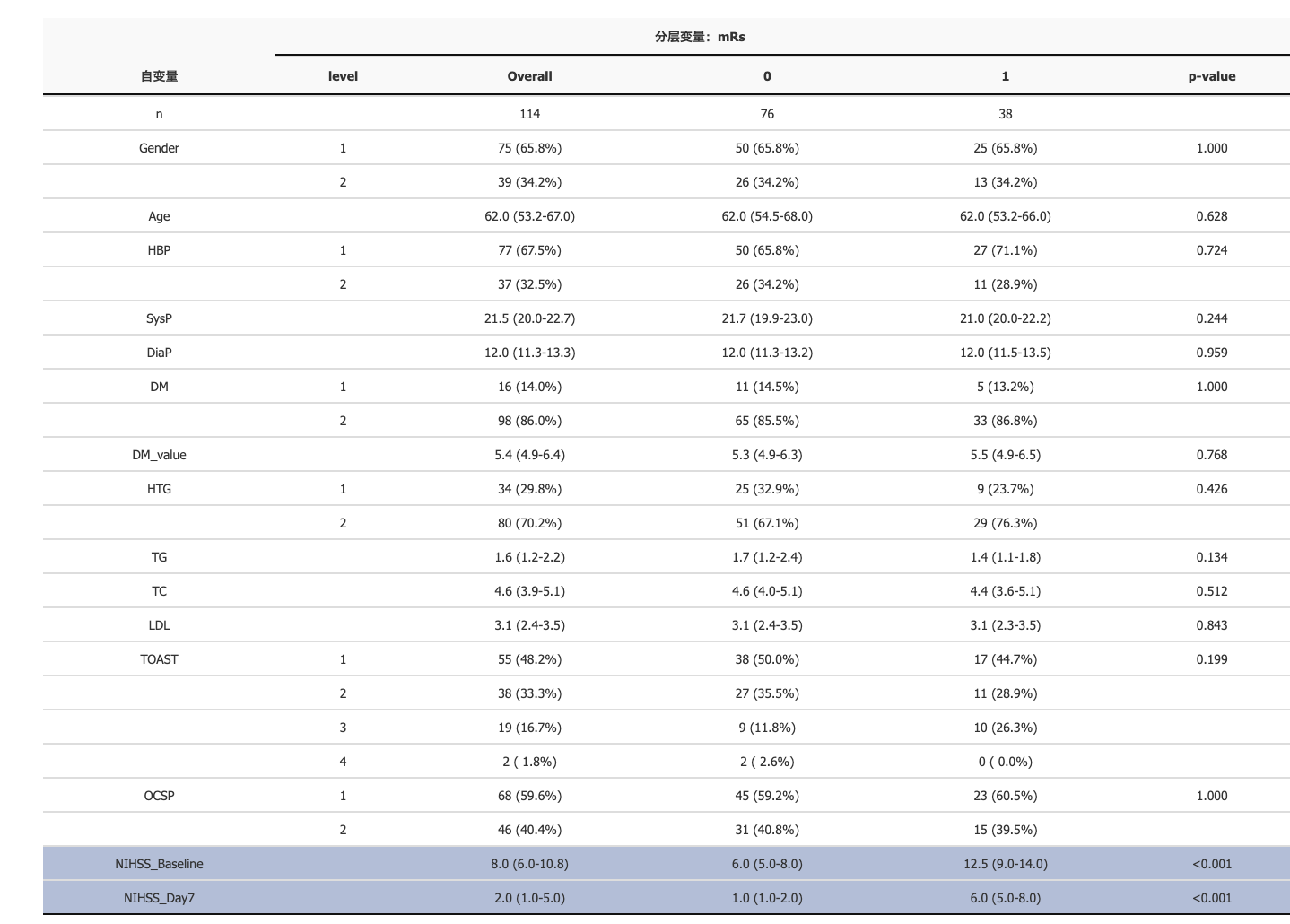
 Supp Table 3. Clinical features in different mRs groups in External Data II**

**Supp Table 4. Single-factor analysis in GLM and GAM in External Data I**


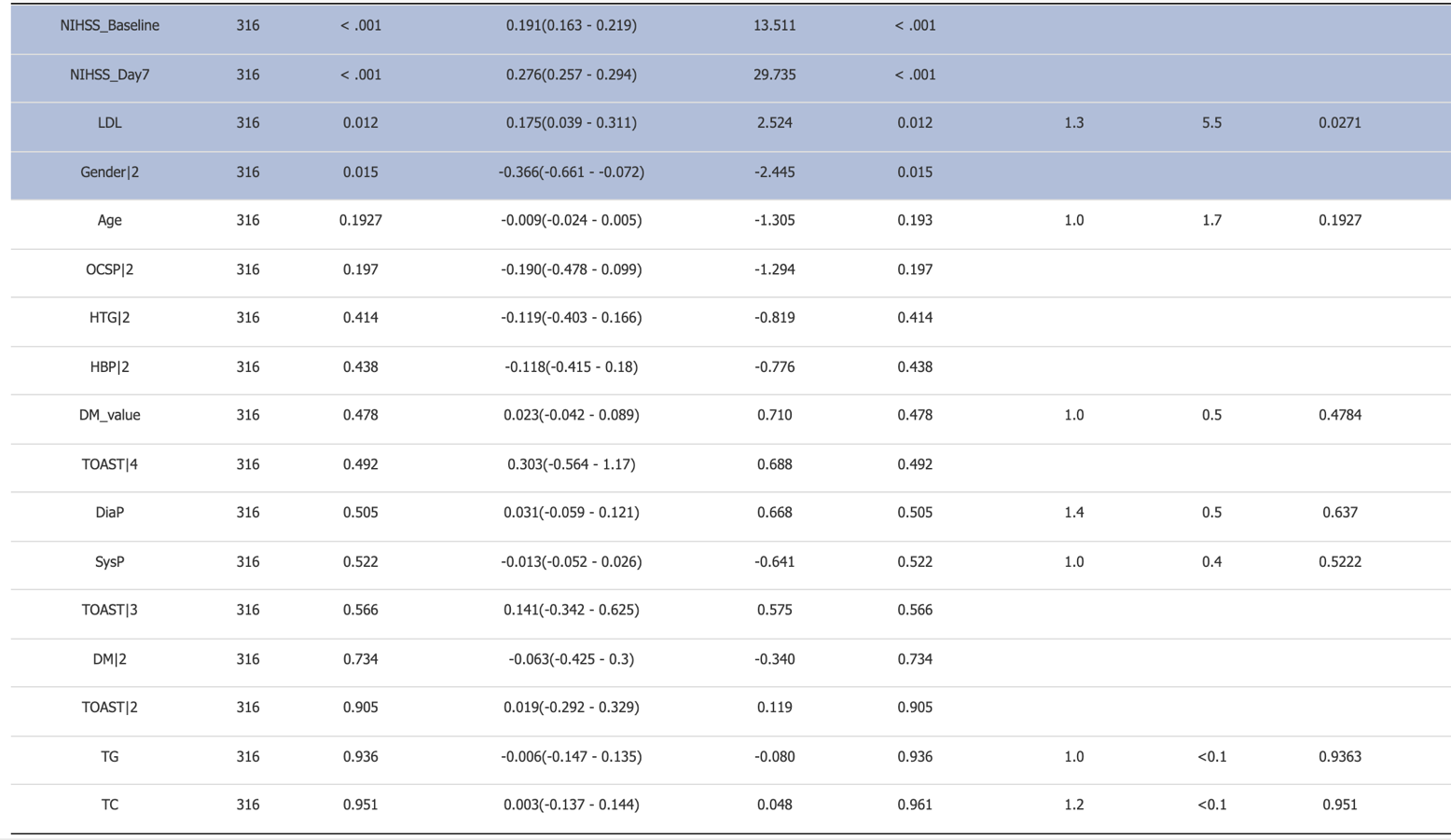

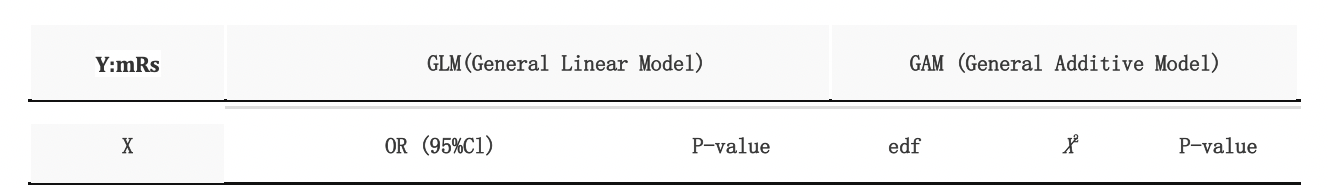


**Supp Table 5. Univariable Logistic analysis in External Data I**


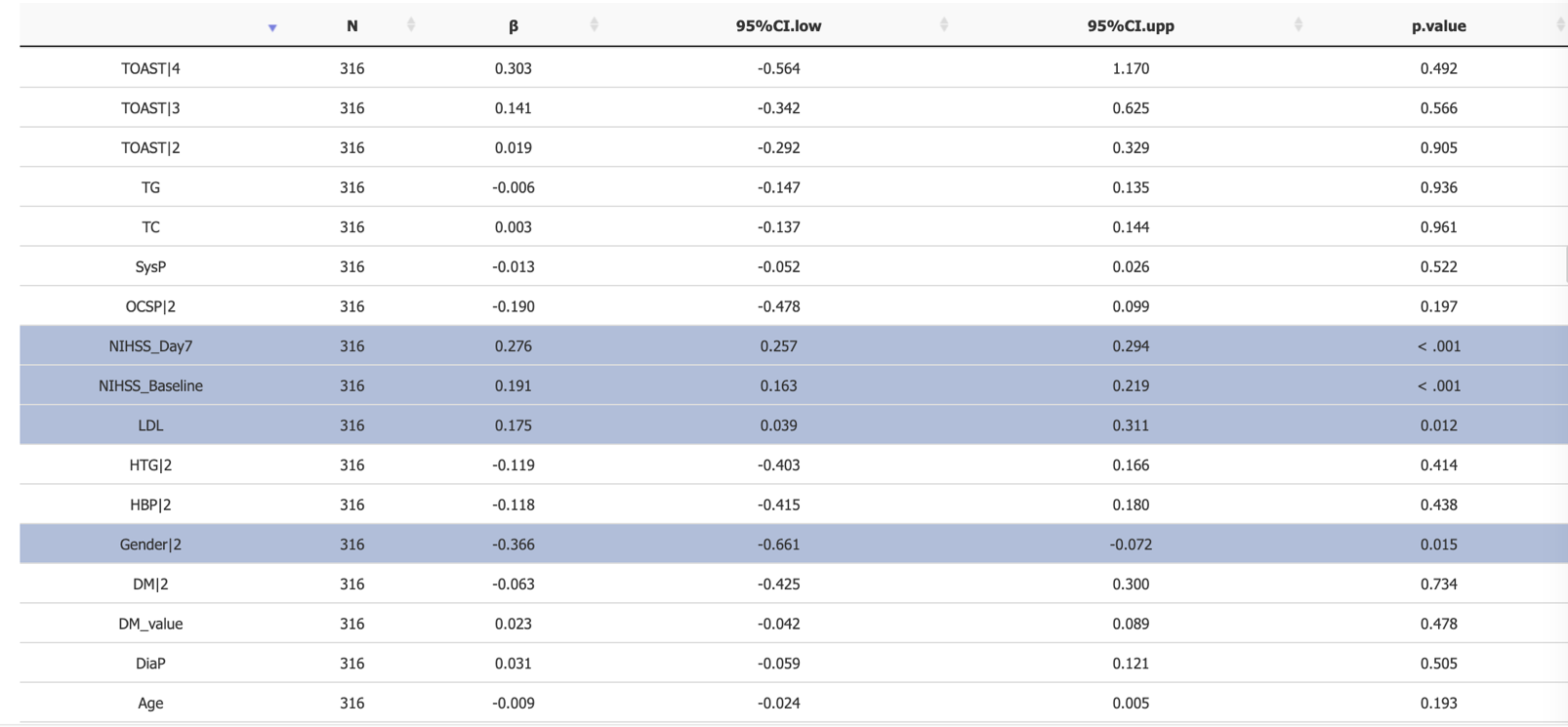


**Supp Table 6. Multivariable Logistic analysis in External Data I**


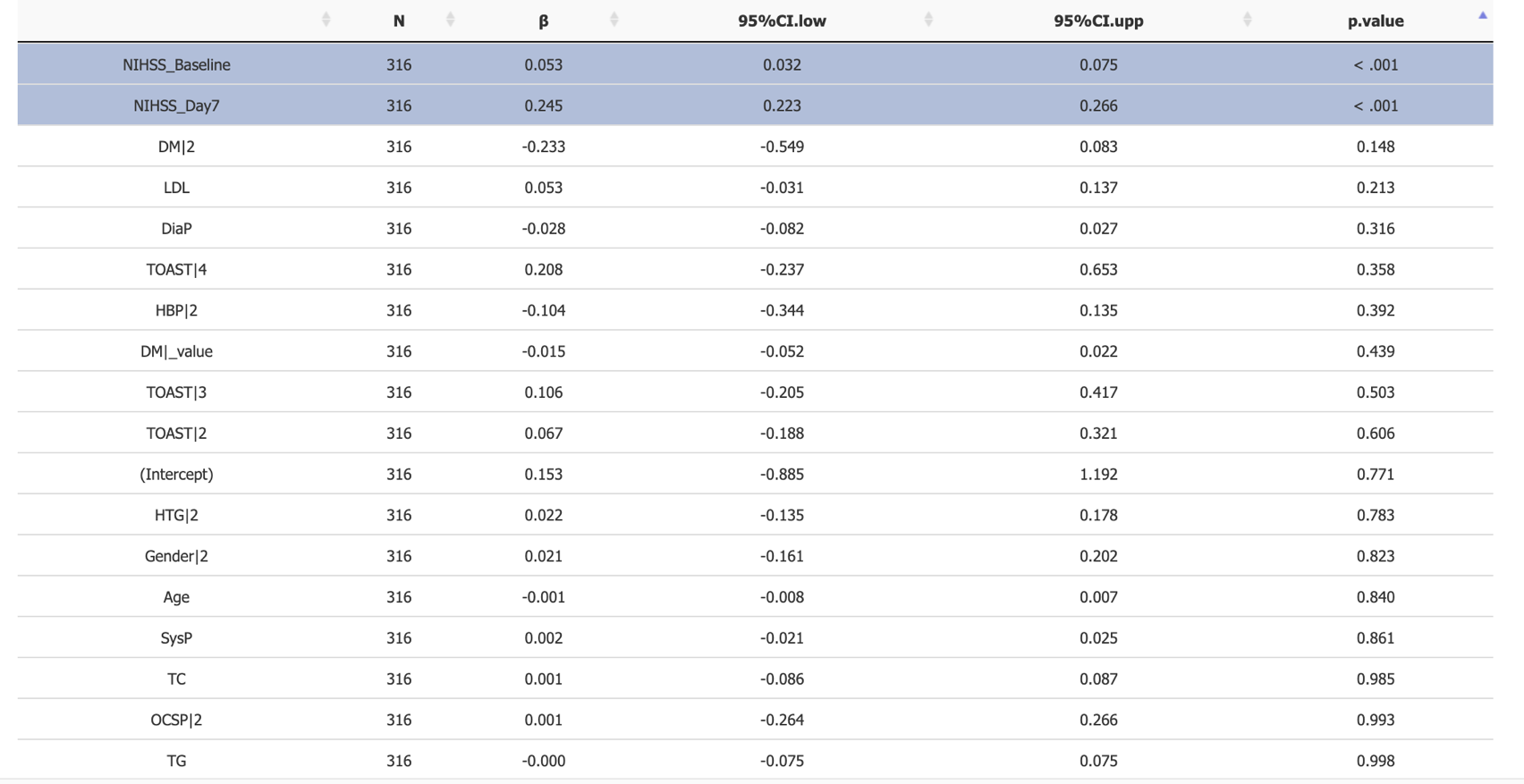


**Supp Table 7 Single-factor analysis in GLM and GAM in External Data II**


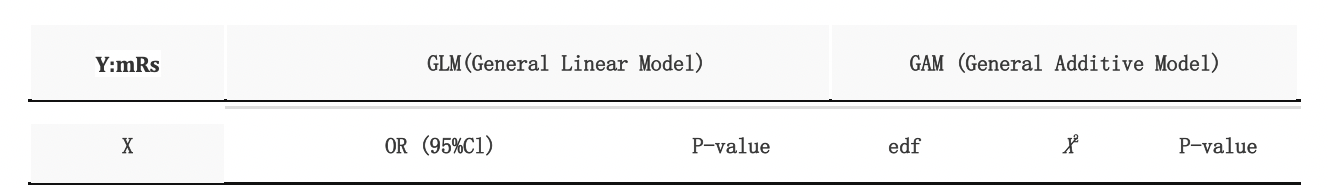

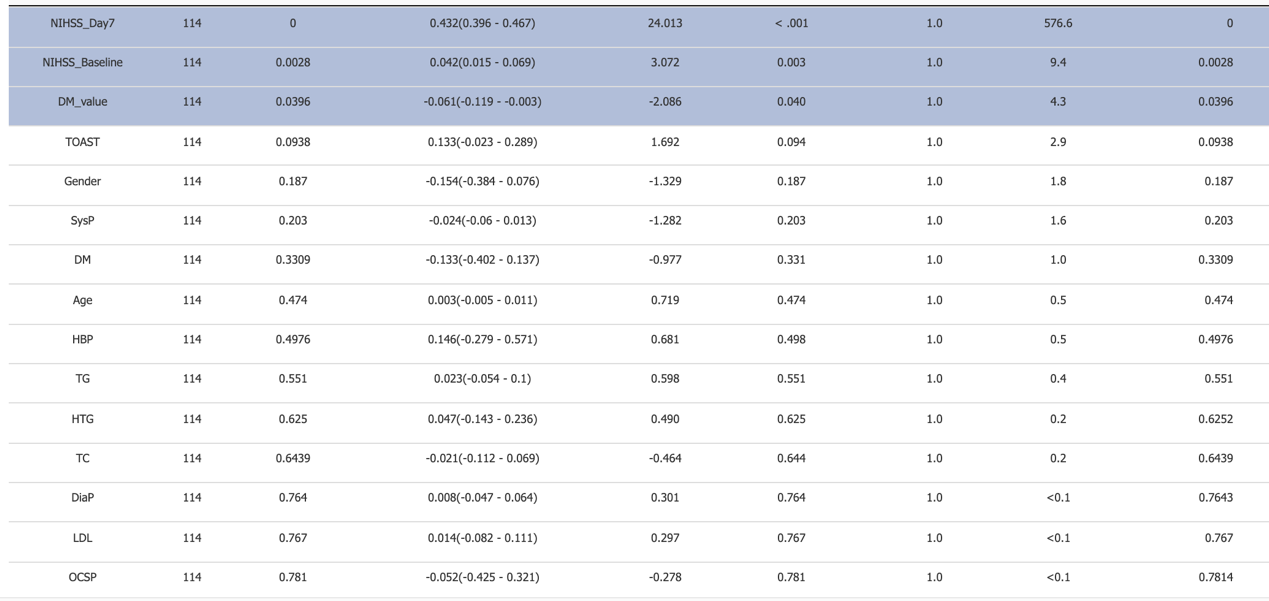


**Supp Table 8 Univariable Logistic analysis in External Data II**

**
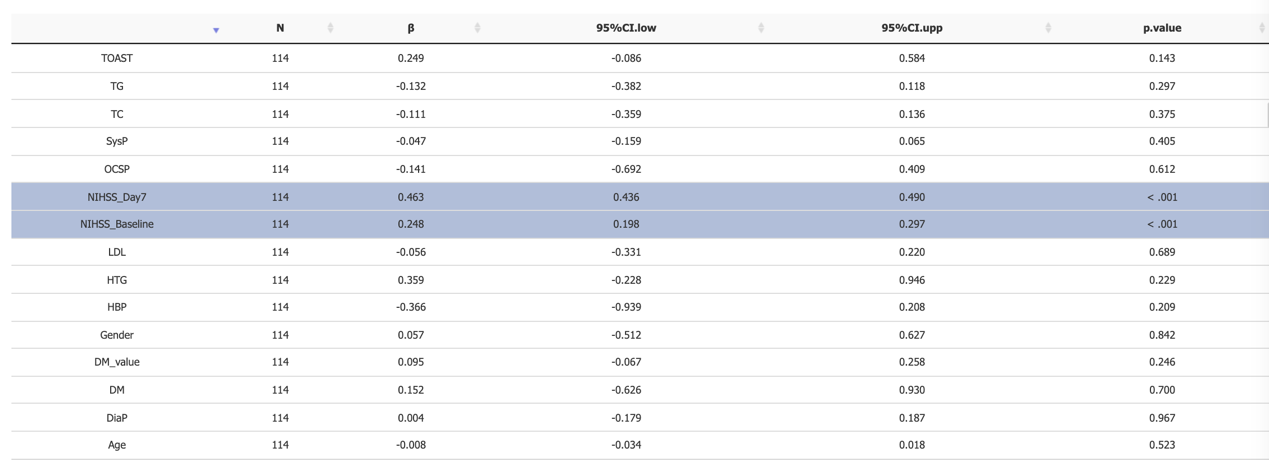
**

**Supp Table 9 Multivariable Logistic analysis in External Data II**

**
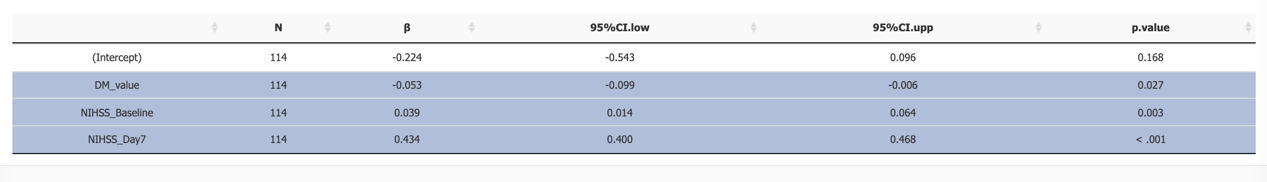
**


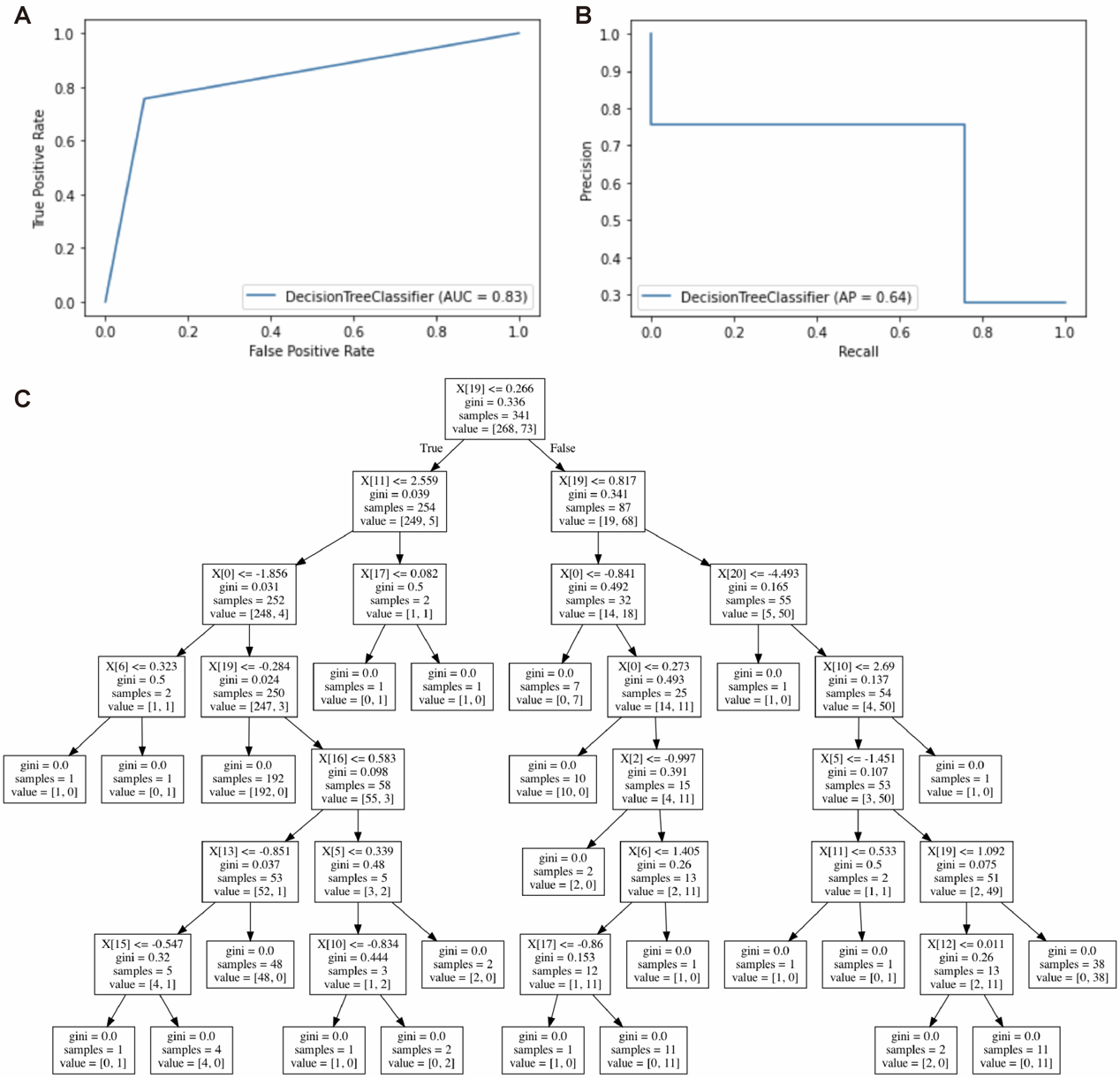


**Supp Figure 1.** **Predictive ability of DecisionTreeClassifier Model**. (A,B) AUC and an AP value of ROC curve for the decision tree classifier (C). The procedure for decision tree analysis shown by Graphviz.


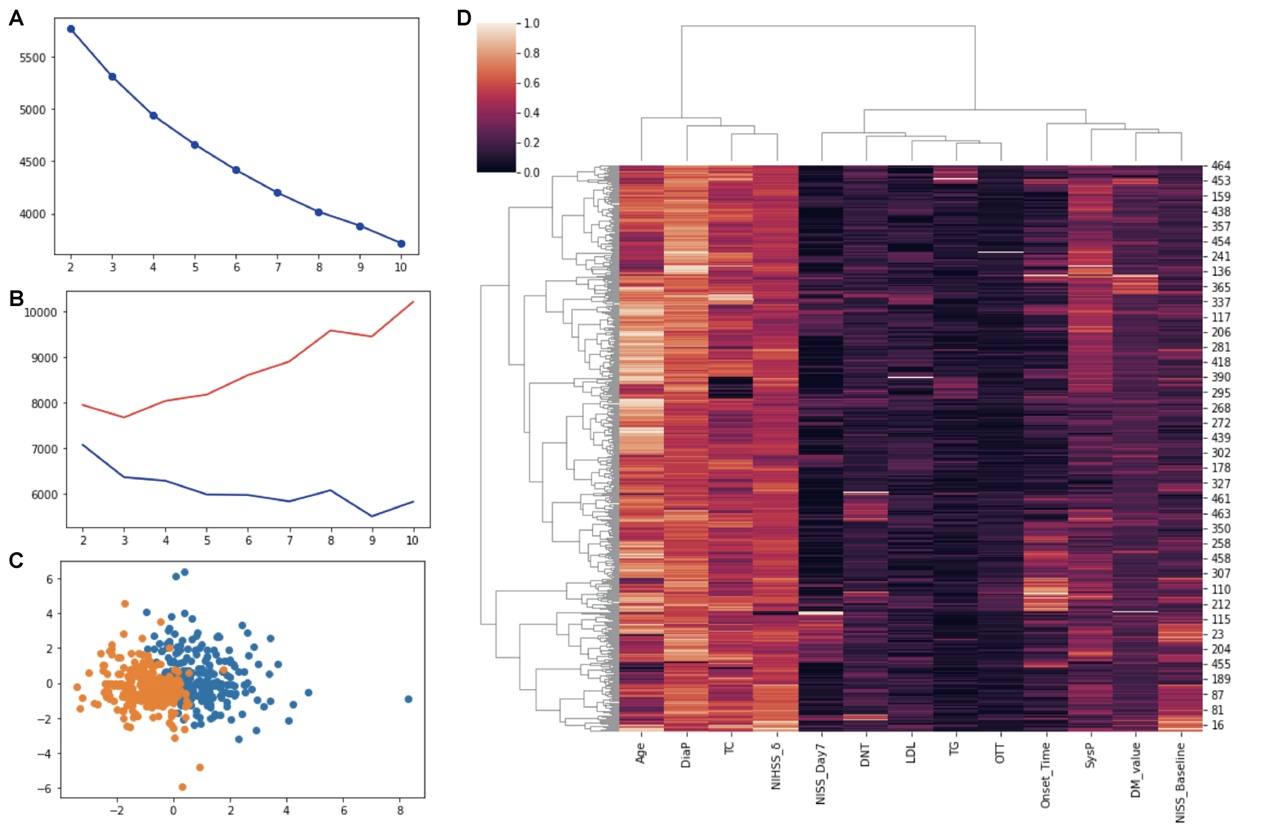


**Supp Figure 2.** The inertia and silhouette score with matplotlib for the **K-means cluster map** (A-C). The cluster map for clinical features drawn with seaborn. The different features were clustered with Agglomerative Clustering method (D).


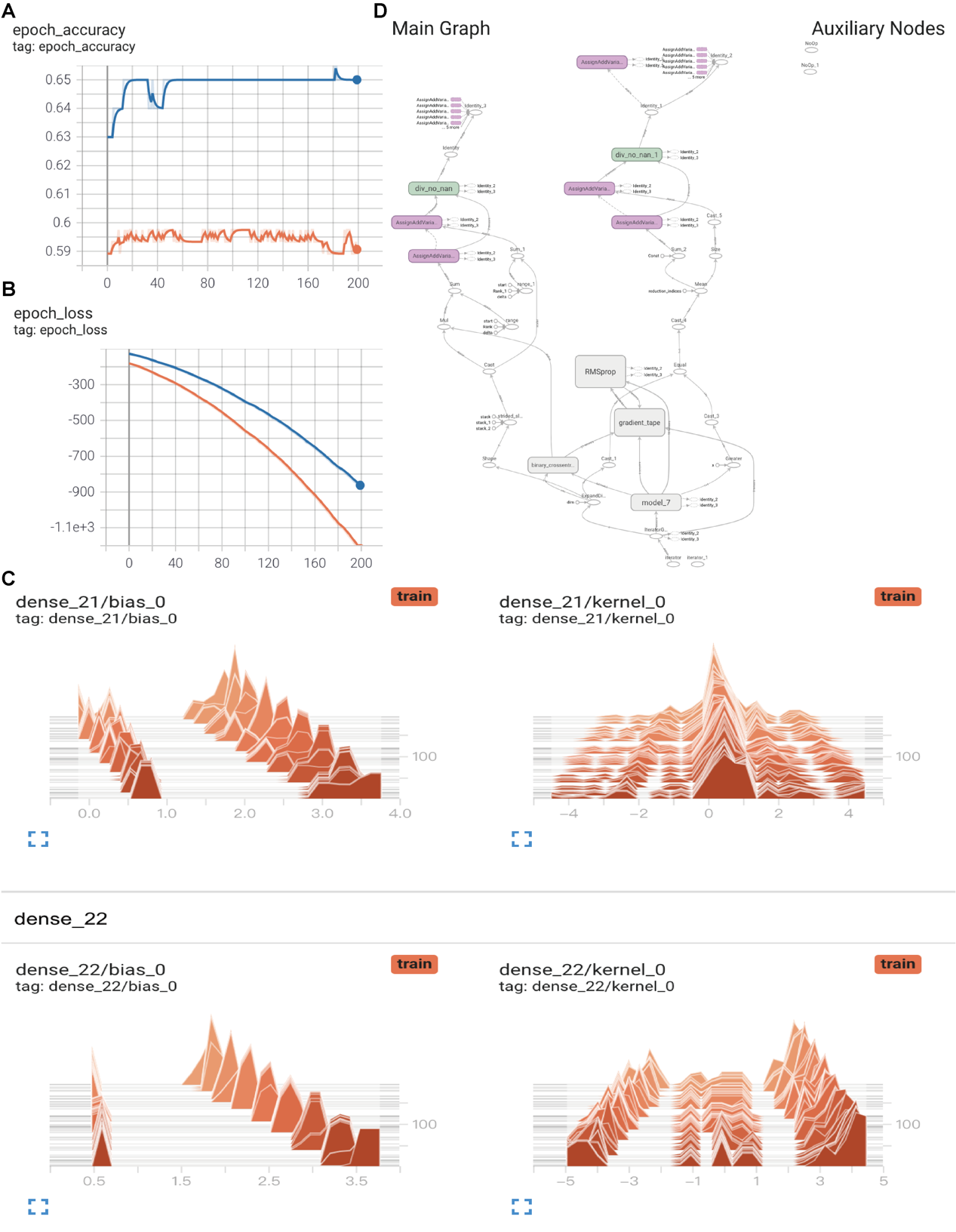


**Supp Figure 3.** The multiple entry model illustrated by TensorBoard of **deep neural network**. A, Kernel training; B, epoch accuracy increased with training; C, epoch loss decreased with training.


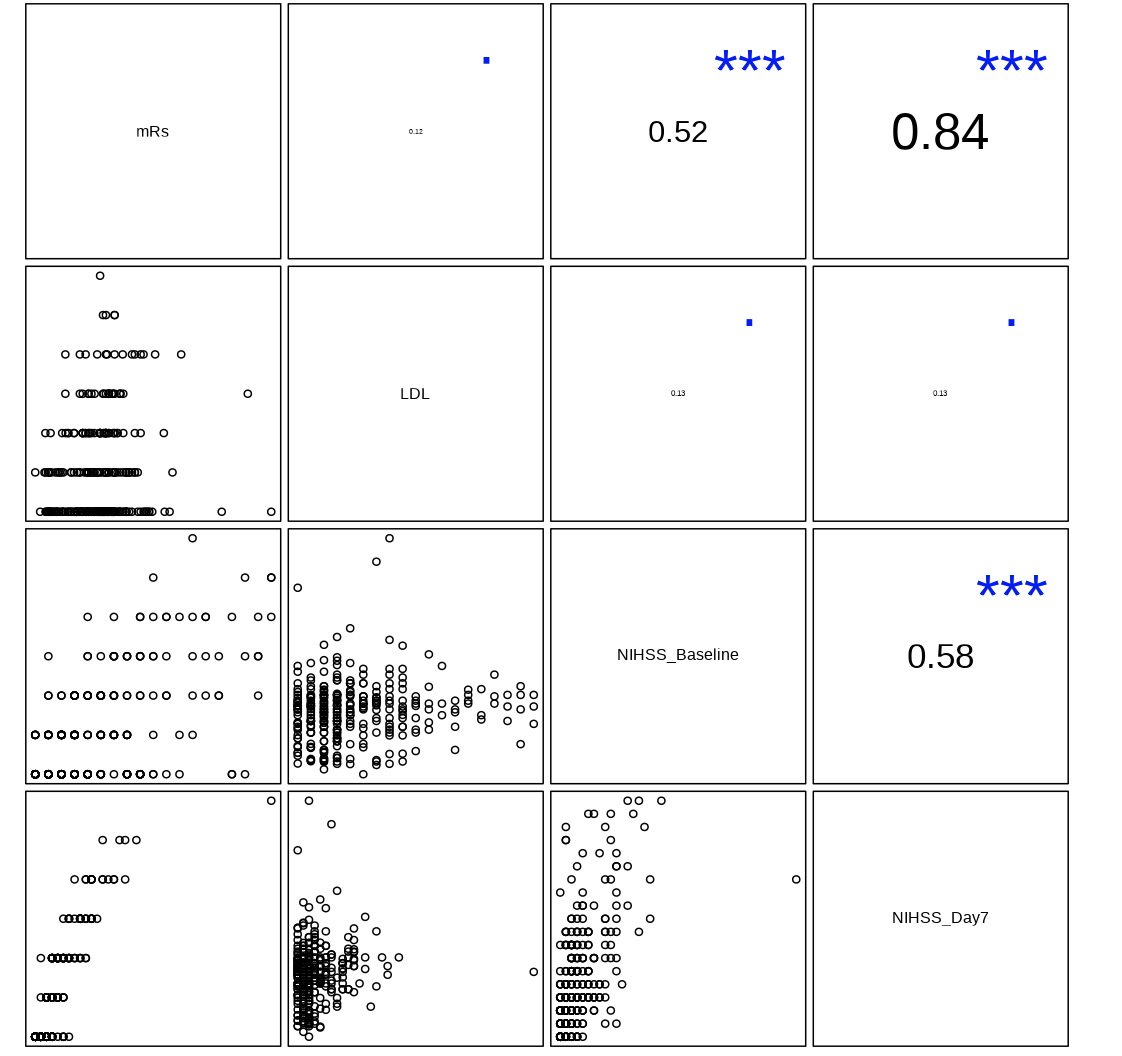


Supp Figure 4. The correlational map between mRs and risk factors based on single-factor ANOVA in External I. The mRs has a positive correlation with LDL, NIHSS_Day7 and NIHSS_Baseline.


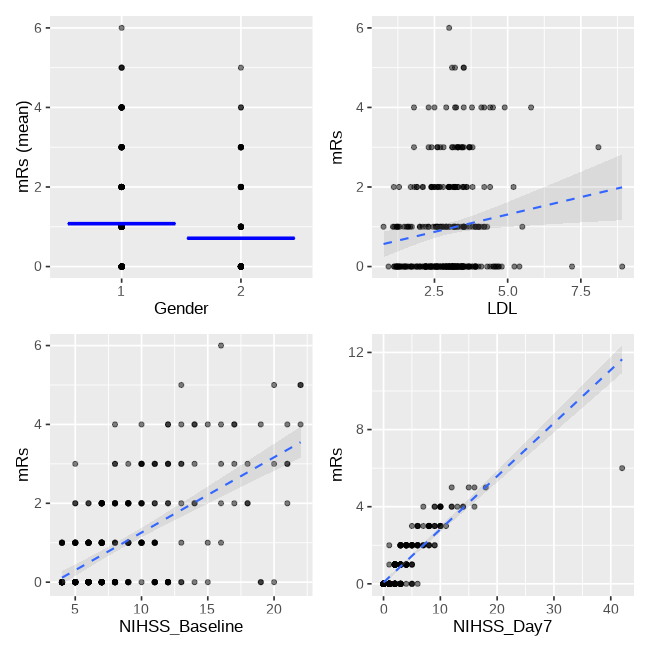


Supp Figure 5.The scatter plot between mRs and risk factors based on single-factor ANOVA in External I.


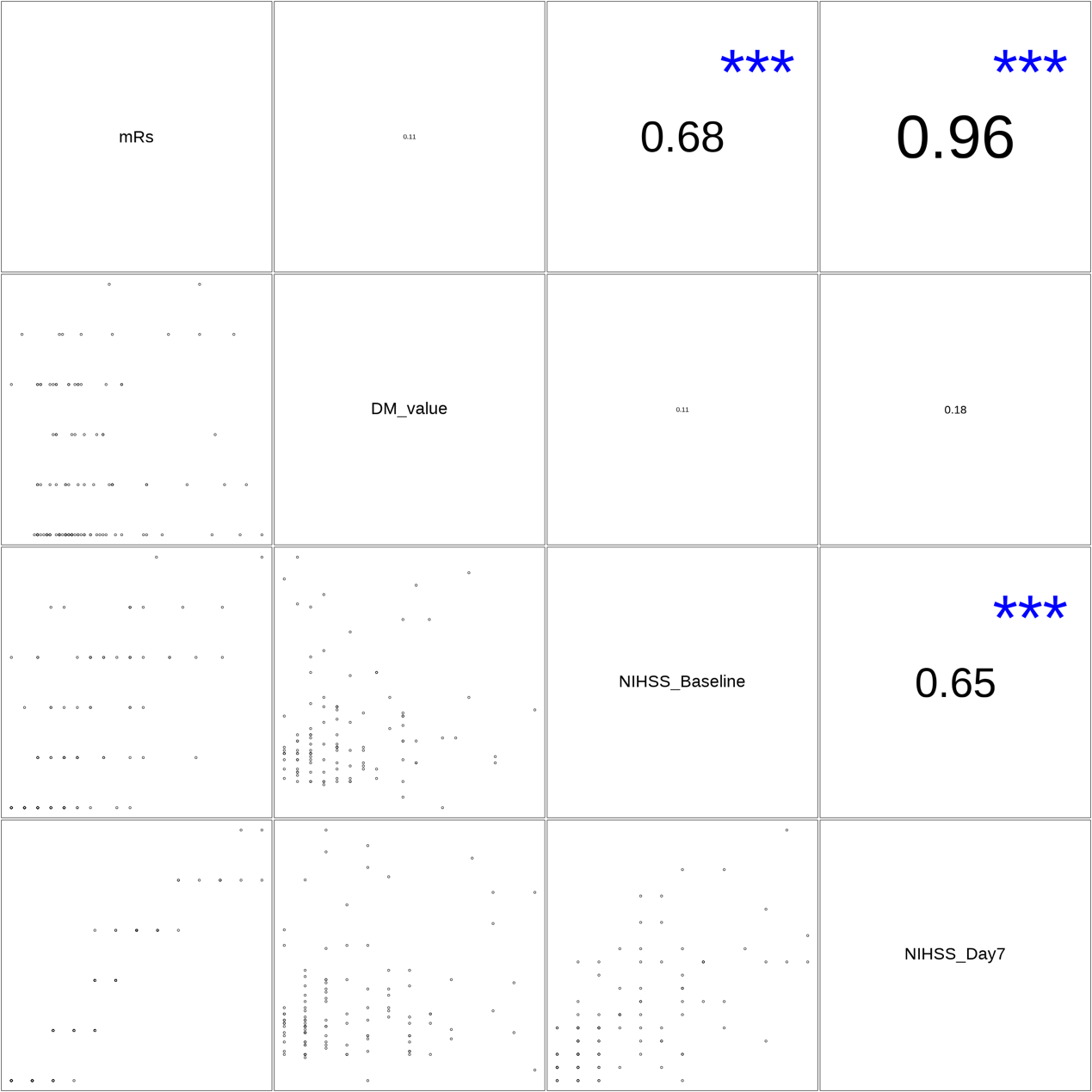


Supp Figure 6. The correlational map between mRs and risk factors based on single-factor ANOVA in External II. The mRs has a positive correlation with DM_value, NIHSS_Day7 and NIHSS_Baseline.


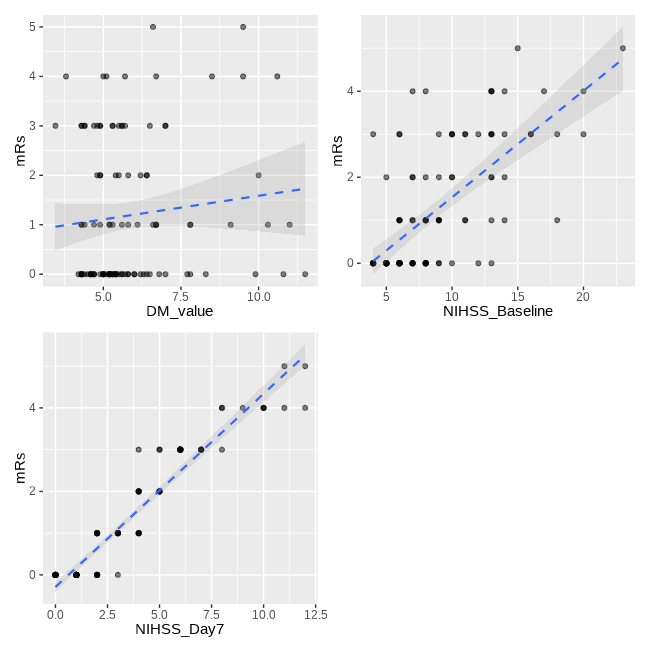


Supp Figure 7.The scatter plot between mRs and risk factors based on single-factor ANOVA in External II.


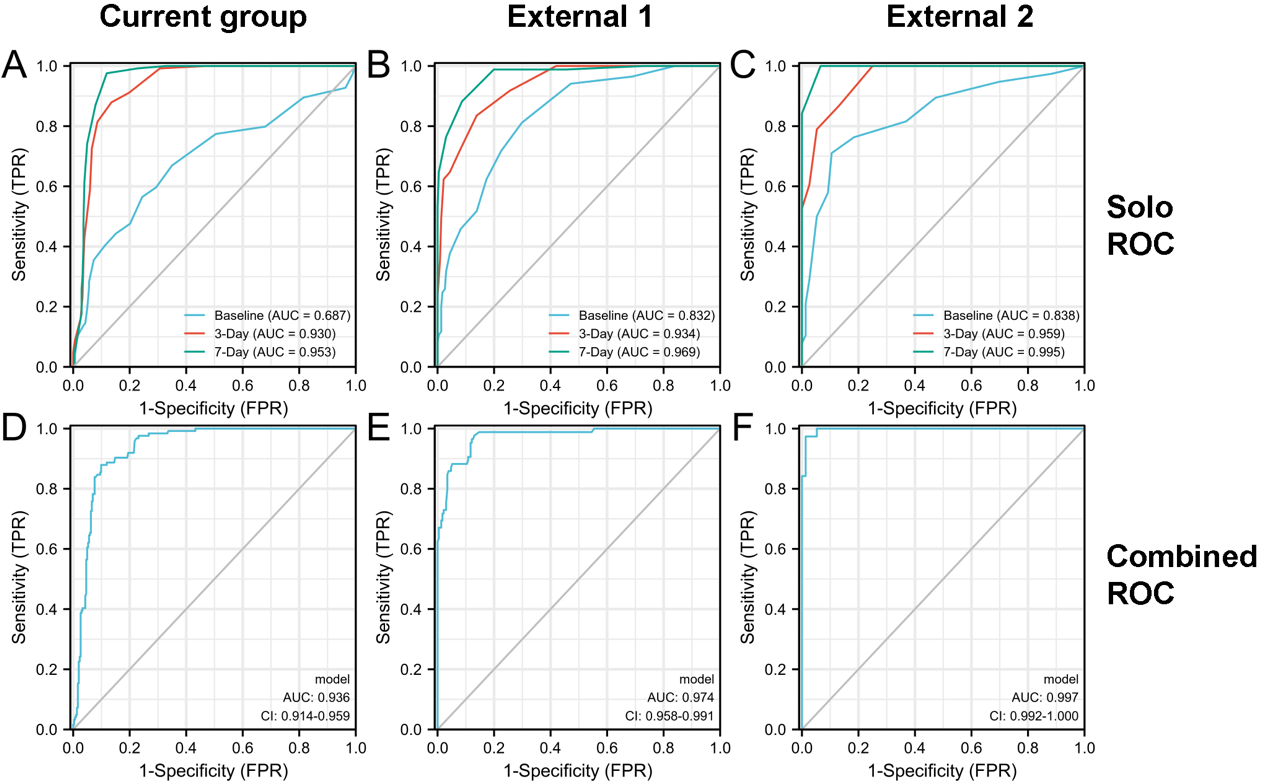


Supp Figure 8. Comparison of the NIHSS parameters for the Prediction of Outcomes. Solo ROC curve for baseline NIHSS, 3-day NIHSS and 7-day NIHSS to predict the MRS at three months post onset (A-C). The AUC value in three groups of combined baseline NIHSS, 3-day NIHSS and 7-day NIHSS, which indicated a strong prediction ability (D-F).
